# Supplementary material for: Plasma Copper Concentration Is Associated with Cardiovascular Mortality in Male Kidney Transplant Recipients
Source: Antioxidants (Basel). 2023 Feb 10;12(2):454. doi: 10.3390/antiox12020454 (PMC9952822; doi:10.3390/antiox12020454)
Supplement: Supplementary file 1 [file antioxidants-12-00454-s001.zip › antioxidants-2175844-supplementary.pdf]

antioxidants

MDPI

Supplemental material

Plasma copper concentration is associated with cardiovascular mortality in male kidney transplant recipients

Manuela Yepes-Calderón[1,*], Daan Kremer[1], Adrian Post[1], Camilo G. Sotomayor[1,2], Ulrike Seidel[3], Patricia Huebbe[3], Tim J. Knobbe[1], Kai Lüersen[3], Michele F. Eisenga[1], Eva Corpeleijn[4], Martin H. de Borst[1], Gerjan J. Navis[1], Gerald Rimbach[3], Stephan J. L. Bakker[1]

[1] Department of Internal Medicine, Division of Nephrology, University Medical Center Groningen, Groningen, The Netherlands.
[2] Institute of Biomedical Sciences, Faculty of Medicine, University of Chile, Santiago, Chile.
[3] Institute of Human Nutrition and Food Science, University of Kiel, Kiel, Germany.
[4] Department of Epidemiology, University Medical Center Groningen, Groningen, The Netherlands.

* Correspondence: m.yepes.calderon@umcg.nl; Tel.: +31 061 6654 888

1. Table of content.

- **Table S1.** Baseline characteristics of male KTR……………………………………..….3
- **Table S2.** Baseline characteristics of female KTR. ………………………………….5
- **Table S3.** Effect-modification of pre-specified baseline characteristics on the associations of plasma copper concentration with cardiovascular mortality in KTR………..………………………………………………………………………….7
- **Table S4.** Association of plasma copper concentration with all-cause mortality, non-cardiovascular mortality and graft failure in KTR…..………………..…..…..…8
- **Table S5.** Sensitivity analyses of the association of plasma copper concentration with risk of cardiovascular mortality in male KTR, after exclusion of patients according to different criteria…………………..………………..….........…………9

Table S1. Baseline characteristics of male KTR.

	Tertiles of plasma copper concentration[π]			$p^{¥}$
	**Tertile 1**	**Tertile 2**	**Tertile 3**	
n	126	121	123	—
Plasma copper concentration, µmol/L	12.12 (11.02–12.75)	14.64 (14.01–15.11)	17.31 (16.37–18.88)	—
**Demographic and body composition**				
Age, years	50 ± 13	54 ± 12	55 ± 13	0.002
Caucasian ethnicity, *n* (%)	124 (98)	121 (100)	123 (100)	0.33
Body mass index, kg/m^2	25.5 ± 3.7	26.8 ± 4.3	27.0 ± 4.5	0.01
Waist circumference, cms[a]	97.5 ± 13	102 ± 12	104 ± 14	0.001
**Renal allograft function**				
eGFR, mL/min/1.73 m^2	53 ± 20	52 ± 19	54 ± 21	0.58
Proteinuria, *n*[b] (%)[b]	31 (25)	38 (31)	32 (26)	0.46
**Renal transplant**				
Preemptive transplantation, *n* (%)	21 (17)	17 (14)	11 (9)	0.19
Dialysis duration before transplantation, months	20 (5–49)	26 (10–51)	28 (11–50)	0.26
Living donor, *n* (%)	48 (38)	36 (30)	43 (35)	0.38
Donor age, years [c]	44 ± 15	43 ± 16	43 ± 16	0.69
Donor sex (male), *n* (%)[d]	55 (44.4)	54 (45.4)	60 (49.6)	0.69
Time since transplantation, years	5.83 (2.35–12.2)	5.84 (1.73–13.3)	5.28 (1.54–9.99)	0.73
**Immunosuppressive therapy**				
Prednisolone use, *n* (%)	126 (100)	119 (98)	122 (99)	0.22
Calcineurin inhibitor use, *n* (%)	73 (58)	71 (59)	74 (60)	0.94
Proliferation inhibitor use, *n* (%)	98 (78)	104 (86)	105 (85)	0.16
Acute rejection treatment, *n* (%)	39 (31)	32 (26)	41 (33)	0.49
**Cardiovascular history**				
History of cardiovascular disease, *n* (%)	24 (19)	31 (26)	42 (34)	0.03
Previous myocardial infarction, *n* (%)	4 (3)	7 (6)	12 (10)	0.10
Previous cerebrovascular event, *n* (%)	1 (1)	3 (2)	6 (5)	0.13
Previous vascular intervention, *n* (%)	11 (9)	18 (15)	18 (15)	0.26
Systolic blood pressure, mmHg	136 ± 15	139 ± 17	137 ± 17	0.43
Diastolic blood pressure, mmHg-	84 ± 11	84 ± 11	85 ± 10	0.61
Antihypertensive use, *n* (%)	109 (87)	113 (93)	110 (89)	0.20
Total cholesterol, mmol/L	4.73 ± 0.91	5.09 ± 1.15	5.15 ± 1.13	0.004

Low-density lipoprotein-cholesterol, mmol/L[b]	2.70 ± 0.75	3.01 ± 1.01	3.10 ± 0.94	0.001
High-density lipoprotein-cholesterol, mmol/L[b]	1.25 ± 0.41	1.30 ± 0.42	1.22 ± 0.38	0.24
Triglycerides, mmol/L	1.85 ± 0.91	2.02 ± 1.26	2.01 ± 1.19	0.42
Statin use, *n* (%)	67 (53)	68 (56)	60 (49)	0.51
Diabetic subjects, *n* (%)	19 (15)	27 (22)	39 (32)	0.01
Hemoglobin, mmol/L	8.41 (1.07)	8.41 (1.03)	8.41 (1.14)	0.99
**Inflammation and oxidative stress**				
Leukocyte count, 10^9/L[b]	8.05 (6.60–9.55)	7.40 (6.40–9.10)	7.70 (6.35–9.65)	0.34
High-sensitivity C-reactive protein, nmol/L[f]	11.02 (4.72–22.03)	25.18 (12.59–49.57)	77.11 (25.18–141.63)	<0.001
Plasma malondialdehyde, µmol/L[g]	2.57 (2.02–3.54)	2.27 (1.78–4.04)	2.50 (2.01–4.28)	0.41
**Lifestyle**				
Smoking behavior, *n* (%)h				0.76
Current	17 (14)	17 (14)	18 (16)	
Previous	52 (44)	60 (50)	47 (42)	
Never	49 (42)	42 (35)	46 (41)	
Alcohol intake >30g/day, *n* (%)[i]	4 (4)	9 (8)	9 (8)	0.28
SQUASH score, minutes/week × intensity	5805 (2528–7946)	6240 (3840–10440)	4720 (1320–8610)	0.007

[π]Tertile 1: <0.86 mg/L. Tertile 2: 0.86-1.00 mg/L, Tertile 3: >1.00 mg/L. [¥]Differences were tested by ANOVA for continuous variables with normal distribution, Kruskal–Wallis test for continuous variables with non–normal distribution and by $\chi 2$ test for categorical variables. Data available in [a]360, [b]369, [c]363, [d]364, [e]353, [f]350, [g]366, [h]346 and [i]337 KTR. KTR, kidney transplant recipients; eGFR, estimated glomerular filtration rate; SQUASH, Short QUestionnaire to ASsess Health–enhancing physical activity.

Table S2. Baseline characteristics of female KTR.

	Tertiles of plasma copper concentration[π]			p[¥]
	Tertile 1	Tertile 2	Tertile 3	
n	97	96	97	—
Plasma copper concentration, µmol/L	13.69 (13.06–14.64)	16.68 (16.05–17.31)	20.46 (19.20–23.60)	—
**Demographic and body composition**				
Age, years	54 ± 12	54 ± 13	52 ± 14	0.57
Caucasian ethnicity, *n* (%)	97 (100)	95 (99)	97 (100)	0.33
Body mass index, kg/m^2	26.4 ± 4.9	26.8 ± 5.6	27.5 ± 6.1	0.33
Waist circumference, cms[a]	93 ± 14	94 ± 15	98 ± 18	0.05
**Renal allograft function**				
eGFR, mL/min/1.73 m^2[b]	52 ± 21	50 ± 19	48 ± 21	0.39
Proteinuria, *n* (%)	12 (12)	18 (19)	20 (21)	0.28
**Renal transplant**				
Preemptive transplantation, *n* (%)	17 (18)	17 (18)	20 (21)	0.83
Dialysis duration before transplantation, months	22 (8–47)	26 (11–48)	22 (8–48)	0.73
Living donor, *n* (%)	36 (37)	35 (37)	31 (32)	0.72
Donor age, years [c]	44 ± 15	42 ± 16	43 ± 15	0.63
Donor sex (male), *n* (%)[d]	54 (56)	51 (56)	60 (64)	0.47
Time since transplantation, years	5.02 (2.15–12.5)	5.90 (2.72–12.1)	4.48 (1.62–12.3)	0.52
**Immunosuppressive therapy**				
Prednisolone use, *n* (%)	96 (99)	94 (98)	96 (99)	0.70
Calcineurin inhibitor use, *n* (%)	57 (59)	48 (50)	59 (61)	0.27
Proliferation inhibitor use, *n* (%)	81 (84)	82 (85)	79 (81)	0.76
Acute rejection treatment, *n* (%)	16 (17)	23 (24)	22 (23)	0.40
**Cardiovascular history**				
History of cardiovascular disease, *n* (%)	15 (16)	22 (23)	24 (25)	0.24
Previous myocardial infarction, *n* (%)	3 (3)	3 (3)	3 (3)	0.99
Previous cerebrovascular event, *n* (%)	5 (5)	6 (6)	3 (3)	0.55
Previous vascular intervention, *n* (%)	6 (6)	9 (9)	3 (3)	0.20
Systolic blood pressure, mmHg	133 ± 17	135 ± 20	133 ± 19	0.80
Diastolic blood pressure, mmHg-	80 ± 10	82 ± 12	80 ± 10	0.33
Antihypertensive use, *n* (%)	81 (84)	82 (85)	85 (88)	0.72
Total cholesterol, mmol/L	5.22 ± 1.11	5.35 ± 1.23	5.44 ± 1.20	0.42

Low-density lipoprotein-cholesterol, mmol/L	2.95 ± 0.96	3.06 ± 0.94	3.15 ± 1.02	0.36
High-density lipoprotein-cholesterol, mmol/L	1.52 ± 0.52	1.58 ± 0.50	1.56 ± 0.53	0.72
Triglycerides, mmol/L	1.82 ± 0.83	1.85 ± 0.85	1.98 ± 0.93	0.39
Statin use, *n* (%)	54 (56)	56 (58)	46 (47)	0.28
Diabetic subjects, *n* (%)	21 (22)	22 (23)	28 (29)	0.46
Hemoglobin, mmol/L	8.11 (0.91)	7.71 (0.94)	7.70 (0.93)	0.002
**Inflammation and oxidative stress**				
Leukocyte count, 10^9/L[b]	7.50 (6.10–9.30)	7.10 (5.50–8.93)	8.30 (6.60–11.1)	0.01
High-sensitivity C-reactive protein, nmol/L[f]	8.57 (3.81–15.05)	15.24 (8.57–35.24)	40.00 (18.57–95.71)	<0.001
Plasma malondialdehyde, μmol/L[b]	2.62 (2.07–3.66)	2.50 (1.90–3.87)	2.64 (1.90–3.61)	0.77
**Lifestyle**				
Smoking behavior, *n* (%)h				0.93
Current	9 (10)	9 (10)	8 (9)	
Previous	44 (45)	40 (43)	42 (45)	
Never	38 (42)	45 (48)	43 (46)	
Alcohol intake >30g/day, *n* (%)[h]	3 (3)	1 (1)	2 (2)	0.87
SQUASH score, minutes/week × intensity	5040 (2040–6870)	4560 (2130–6611)	4560 (1800–6960)	0.68

[π]Tertile 1: <0.97 mg/L. Tertile 2: 0.97-1.13 mg/L, Tertile 3: >1.13 mg/L. [¥]Differences were tested by ANOVA for continuous variables with normal distribution, Kruskal–Wallis test for continuous variables with non–normal distribution and by χ2 test for categorical variables. Data available in [a]278, [b]289, [c]279, [d]281, [e]282, [f]274, [g]275 and [h]261 KTR. KTR, kidney transplant recipients; eGFR, estimated glomerular filtration rate; SQUASH, Short QUestionnaire to ASsess Health–enhancing physical activity.

Table S3. Effect-modification of pre-specified baseline characteristics on the associations of plasma copper concentration with cardiovascular mortality in KTR.

**Pre-specified potential effect-modifiers**	p interation
Sex, male	0.01
Body mass index, kg/m^2	0.69
Systolic blood pressure, mmHg	0.97
Total cholesterol, mmol/L	0.64
Low-density lipoprotein-cholesterol, mmol/L	0.47
High-density lipoprotein-cholesterol, mmol/L	0.08
High-sensitivity C-reactive protein, nmol/L	0.16
Leukocyte count, 10^9/L	0.48

Cox-proportional hazards regression analyses were performed with the inclusion of multiplicative interaction terms. KTR, kidney transplant recipients.

Table S4. Association of plasma copper concentration with all-cause mortality, non-cardiovascular mortality and graft failure in KTR.

	Copper plasma concentration (ln, per 1-SD increase)								
	Overall KTR			Male KTR			Female KTR		
	HR	95% CI	*p*	HR	95% CI	*p*	HR	95% CI	*p*
**All–cause mortality**									
Crude	1.16	0.99–1.37	0.07	1.39	1.08–1.79	0.01	1.07	0.83–1.39	0.59
Model 1	1.15	0.96–1.39	0.14	1.22	0.94–1.58	0.13	1.09	0.83–1.44	0.53
Model 2	1.18	0.99–1.43	0.07	1.27	0.98–1.62	0.07	1.10	0.82–1.46	0.53
Model 3	1.16	0.96–1.39	0.12	1.27	0.99–1.64	0.06	1.09	0.82–1.44	0.55
Model 4	1.11	0.92–1.33	0.30	1.17	0.91–1.50	0.22	1.04	0.78–1.38	0.80
Model 5	1.13	0.95–1.35	0.18	1.23	0.95–1.59	0.12	1.04	0.80–1.36	0.76
Model 6	1.12	0.91–1.38	0.27	1.15	0.87–1.51	0.33	1.11	0.79–1.55	0.55
**Non-cardiovascular mortality**									
Crude	1.04	0.84–1.29	0.72	1.03	0.74–1.43	0.88	1.06	0.77–1.44	0.74
Model 1	0.98	0.77–1.24	0.85	0.88	0.64–1.24	0.48	1.08	0.77–1.51	0.67
Model 2	1.01	0.80–1.23	0.90	0.97	0.69–1.36	0.85	1.07	0.75–1.52	0.73
Model 3	0.98	0.78–1.24	0.88	0.95	0.69–1.31	0.76	1.07	0.76–1.51	0.69
Model 4	0.95	0.74–1.20	0.65	0.87	0.63–1.21	0.42	1.00	0.72–1.42	0.95
Model 5	0.97	0.77–1.23	0.82	0.90	0.64–1.27	0.55	1.06	0.76–1.49	0.72
Model 6	1.05	0.80–1.37	0.73	0.95	0.66–1.38	0.81	1.13	0.75–1.70	0.57
**Graft failure**									
Crude	0.85	0.68–1.07	0.16	0.86	0.62–1.18	0.35	0.82	0.58–1.18	0.29
Model 1	0.85	0.67–1.08	0.19	0.87	0.62–1.21	0.41	0.79	0.56–1.11	0.17
Model 2	0.84	0.66–1.07	0.15	0.93	0.66–1.33	0.70	0.73	0.51–1.03	0.07
Model 3	0.86	0.68–1.09	0.21	0.88	0.63–1.22	0.44	0.77	0.54–1.09	0.14
Model 4	0.79	0.61–1.00	0.05	0.82	0.58–1.15	0.25	0.71	0.49–1.02	0.06
Model 5	0.82	0.64–1.04	0.09	0.84	0.60–1.18	0.31	0.74	0.51–1.06	0.10
Model 6	0.84	0.65–1.09	0.18	0.81	0.57–1.16	0.25	0.80	0.54–1.18	0.27

Model 1: adjustment for age, sex and BMI. Model 2: model 1 + eGFR, time since transplantation and dialysis time before transplantation. Model 3: model 1 + calcineurin inhibitor use, proliferation inhibitor use. Model 4: model 1 + SBP, history of previous cardiovascular disease and hemoglobin.. Model 5: model 1 + total cholesterol, low-density lipoprotein cholesterol, diabetes history. Model 6: model 1 + SQUASH score + high-sensitivity C-reactive protein. KTR, kidney transplant recipients; ln, natural logarithm, SD, standard deviation; HR; hazard ratio; CI, confidence interval; SQUASH, Short QUestionnaire to ASsess Health–enhancing physical activity.

Supplemental Table 5. Sensitivity analyses of the association of plasma copper concentration with risk of cardiovascular mortality in male KTR, after exclusion of patients according to different criteria.

	Copper plasma concentration (ln, per 1-SD increment)			
	n_{events}	HR	95% CI	*p*
With eGFR <30 mL/min/1.73 m^2	35	2.24	1.42–3.55	0.001
Who died within the 1st year of follow–up	30	1.74	1.10–2.75	0.02
Outside -2 and +2 SD of plasma copper concentration	30	1.83	1.19–2.81	0.01

Cox proportional–hazards regression analyses were performed with adjustment for age and body mass index. KTR, kidney transplant recipients; ln, natural logarithm, SD, standard deviation; HR; hazard ratio; CI, confidence interval; eGFR, estimated glomerular filtration rate.
